# COVID-19 vaccination-related autoimmune hepatitis—a perspective

**DOI:** 10.3389/fphar.2023.1190367

**Published:** 2023-08-03

**Authors:** Consolato M. Sergi

**Affiliations:** ^1^ AP Division, Pathology Laboratories, Children’s Hospital of Eastern Ontario, University of Ottawa, Ottawa, ON, Canada; ^2^ Department of Laboratory Medicine and Pathology, University of Alberta, Edmonton, AB, Canada

**Keywords:** vaccination, COVID-19, autoimmune hepatitis, myocarditis, liver, adverse reaction, bioinformatics, public health

## Abstract

Autoimmune hepatitis (AIH) is the inflammation of the liver with clear-cut interface hepatitis and piecemeal necrosis located at the boundary between portal areas and periportal hepatocytes, and characterized by autoimmunity to hepatocytes with an increase in the antinuclear antibody. After the disastrous SARS-CoV-2 pandemic flagellated several countries, several vaccines have been commercialized and have become a ground for social responsibility. The mRNA vaccines, issued by Pfizer-BioNTech (BNT162b2) and Moderna (mRNA-1273), do not use prebuilt viruses to supply the antigen in the subject’s body and are not perfect but have been useful in tackling the pandemic. Nevertheless, both myocarditis and AIH have been reported as side effects of the vaccination programs in addition to thromboembolic events. Here, we explore this topic and give a data-based perspective, gathering a comparison between the titin protein of the sarcomere and myocarditis. The isolation of a *Drosophila* gene using the serum from a patient with autoimmune scleroderma recognized an epitope on chromosomes (condensed mitotic form) in both human cultured cells and early *Drosophila* embryos. It revealed that this gene encodes a *Drosophila* homolog of the vertebrate titin (D-Titin). Moreover, anti-titin antibodies have been found in a subset of patients with *myasthenia gravis*, a neuromuscular junction disease that is mostly associated with autoimmune antibodies, such as the anti-acetylcholine receptor antibody. The co-existence of *myasthenia gravis* and autoimmune hepatitis is rare, and a cohort of patients with *myasthenia gravis* anti-titin antibodies seems to be highly relevant. In consideration of these data and the number of patients who may not be symptomatic, we postulated that autoimmune phenomena may not be exceedingly rare, following the administration of mRNA technology-based vaccines, and a balance between pros and cons in administrating boosters is critical.

## Introduction

**FIGURE 1:**
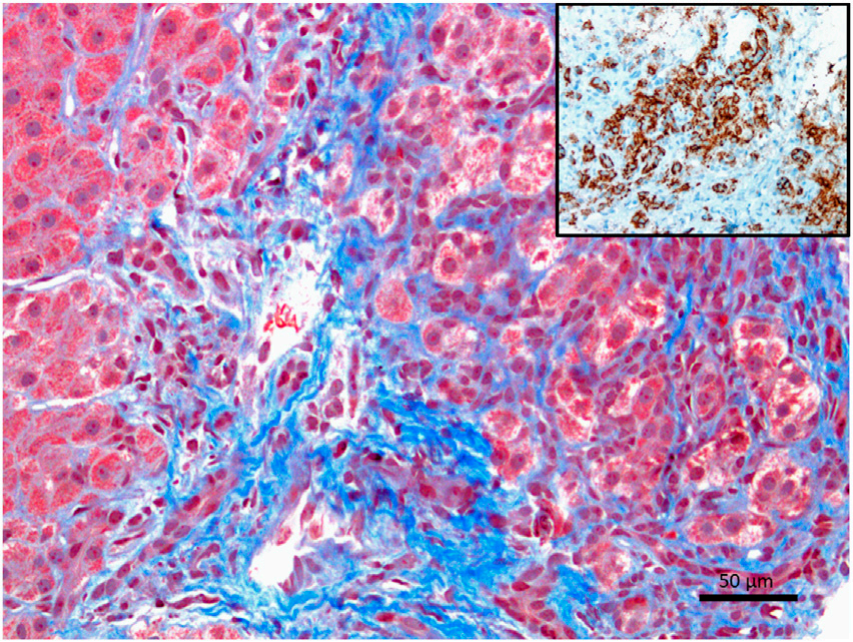
Liver histology of a patient with confirmed autoimmune hepatitis, showing clinical laboratory values, autoantibody positivity, and histopathology, suggestive of autoimmune hepatitis (Masson’s trichrome stain showing an expansion of the portal tract with interface hepatitis, X200). The inset shows an infiltration of plasma cells. CD138, also known as syndecan-1 (SDC1), is a marker useful in histopathology. It is expressed in terminally differentiated plasma cells. CD138 is frequently utilized to highlight plasma cells either by immunohistochemistry or flow cytometry.

Autoimmune hepatitis (AIH) is an inflammation of the liver with clear-cut interface hepatitis and piecemeal necrosis located at the boundary between portal areas and periportal hepatocytes and is characterized by autoimmunity to hepatocytes with an increase in the antinuclear antibody (ANA). A lymph–plasma cellular infiltrate often populates the portal tracts with minimal hepatocytic injury of the central and mid-zonal regions (zones 3 and 2 of the Rappaport) of the liver acinus (Beer and Dienes, 2021; Covelli et al., 2021; Domerecka et al., 2021). The diagnostic criteria of AIH include laboratory findings in addition to the aforementioned histological findings. Alanine aminotransferase and aspartate aminotransferase increased rates, hypergammaglobulinemia, and increased IgG values in the serum are frequently found. In addition, serological abnormalities of specific autoantibodies (antinuclear antibodies [ANA], smooth muscle antibodies [SMA], or anti-liver/anti-kidney microsome 1 [LKM1]) are often present. Although plasma cells are not always present and abundant, their presence corroborates the diagnosis of AIH more substantially than the mononuclear cell infiltrate (Figure 1).

In most cases, the nature of this inflammatory disorder is idiopathic. Still, it is presumed to have an infectious trigger behind the initialization of the inflammatory process of the liver. In some cases, the cause is a virus, which stimulates an inflammatory response, and vitamin D harboring randomized clinical trials has been promising in addition to immunosuppressive therapy (Domerecka et al., 2021; Sergi, 2022a).

### COVID-19 vaccines

After the disastrous SARS-CoV-2 pandemic flagellated several countries, several vaccines have been commercialized and have become a ground for social responsibility (Sergi, 2021; Sergi and Leung, 2021). It may be necessary to start a brief digression at this stage, informing that some mild symptoms may occur with all vaccines. This is due to the local and systemic reactions of the immune system, which seems to be due to a foreign body being inoculated into the body. The mRNA vaccines, issued by Pfizer-BioNTech (BNT162b2) and Moderna (mRNA-1273), do not use prebuilt viruses to inject the antigen in the subject’s body. The technology differs from the traditional methodology in the AstraZeneca vaccine, which uses a customized adenovirus to hold parts of SARS-CoV-2 capable of encouraging the immune response. Traditional vaccination expertise incorporates a hybrid adenovirus–coronavirus assemblage. It stimulates the production of antibodies to both viruses. mRNA vaccines gain attraction because the individual’s immune system generates antibodies more efficiently and effectively. Eventually, the adenovirus deceives or may deceive it. In other words, the immune system may opt not to create antibodies against a partially known pathogenic agent, especially in some subjects. In the United Kingdom, the massive deployment of the AstraZeneca vaccine has only been occasionally associated with some forms of vaccine-induced thrombophilia. It has gained success worldwide with minimal side effects. The production of specific neutralizing antibodies capable of protecting the host and understanding the circulating immune memory for SARS-CoV-2 in case of re-exposure is critical.

In patients who contracted COVID-19, the spike IgG (immunoglobulin G) antibody titer was stable for over 6 months. Moreover, B cells carrying a specific memory and able to produce immunoglobulins were more copious at 6 months than at 1 month after infection. T CD4^+^ and T CD8^+^ lymphocytes specific for SARS-CoV-2 showed a half-life of 3–5 months (Dan et al., 2021). Widge et al. (2021) showed that the developed mRNA-1273 vaccine from Moderna Biotech induced more elevated levels of neutralizing specific antibodies, which decreased slightly over time than other vaccines (Widge et al., 2021). The authors suggested that the mRNA-1273 vaccine can provide lasting and unfluctuating humoral immunity in a way better than that conferred by natural infection (Widge et al., 2021). The risks raised by the skepticism of biotechnology that the host DNA may be mutated remain groundless, but the long-term efficacy has been deeply discussed. Unfortunately, fake news and hesitancy or refusal has been circulating on social media, major newspapers, and television broadcasts. They raised concerns and doubts about the whole process of validation of vaccination programs, and entire cohorts of children may have missed their traditional non-COVID-19 vaccine immunization (Sergi and Leung, 2021). It is important to emphasize that the mRNA cannot cause infection, differently from the virus, since it does not penetrate the cell nucleus.

Furthermore, it cannot integrate into the nuclear DNA of host cells. As a result, the mRNA has an extremely low half-time. Therefore, it needs to be kept at low temperatures for transportation before being inoculated. Following translation, to code proteins, the mRNA must be degraded with its lipid carrier. The mRNA technology may be crucial for developing potential vaccines against several other infectious agents and, probably, against non-infectious diseases and even cancer. It has been predicted that a new personalized vaccine may, in the nearest future, generate precise immune responses when given with a drug adjuvant (e.g., a checkpoint inhibitor) to reduce the tumor size (Chan et al., 2021; Thorp et al., 2022) effectively.

### C19Vax-AIH

An outcry of patient betrayal and break of the Nuremberg code have been divulgated on the internet, raising significant concerns among scientists and laypeople (Thorp et al., 2022). There has been an increasing rate of hesitancy to vaccines against COVID-19 and other vaccines. Vaccines are essential and one of the major steps forward in human medicine since the Vesalius discovery of blood circulation. They are also essential to our society and show how our interaction is critical to developing a functioning society. It is a joint social responsibility to consider vaccination a critical task to reduce the burden for our healthcare systems. On the other hand, there are legitimate questions regarding the process to finalize the approval of some vaccines without appropriate animal testing, as adopted for previous vaccination programs. Although the “warp speed” might have been necessary to stop or limit the pandemic, these perplexities and considerations have diffused and radicalized laymen’s and medical classes’ opinions.

As the COVID-19 pandemic restrictions have been lifted in most countries, it may seem irrelevant looking at liver diseases occurring during an infection or following COVID-19 vaccination and boosters. Despite some drugs being suggested to work well against SARS-CoV-2, vaccines may be considered the only proven defense against this infection. It is usually assumed that vaccine development requires about a decade to develop. Still, with its socio-economic relevance, the loss of lives and the lockdown had needed an emergency path that permitted the development of vaccines at an ultra-rapid speed. Most of these vaccines received approval for mass vaccination programs without proper application of the longtime established guidelines of large clinical trials. The adverse reactions included the local site of injection reaction and mild systemic symptoms like myalgia accompanying fever. In addition to these minor side effects, significant side effects have been reported, ranging from stroke to myocardial infarction and other thromboembolic events. These events seem to have an autoimmune background, but it is unclear. It is well known that molecular mimicry may be the basis of several autoimmune reactions in the human body. Due to its molecular mimicry, the antibodies against the SARS-CoV-2 spike protein may disclose a high affinity for several human proteins and trigger autoimmune phenomena (Sergi, 2022a).

Autoimmune responses after immunization are pretty rare. They occur in less than one per 10,000 of all those who received a vaccine injection. Nevertheless, there seems to be some under-reporting, which has been suggested mostly for myocarditis, pericarditis, or “cardiac discomfort.” Although most cardiac or pericardial reactions have been mild or minimally symptomatic, a proper assessment of cardiac involvement has been hypothesized but unsatisfactorily targeted. It was factually emphasized on the molecular mimicry with the titin protein but poorly evaluated. The reasons may have been outside the political spectrum, from an economic point of view, because cardiac MRI (magnetic resonance imaging) may not be available in all countries and centers.

**FIGURE 2:**
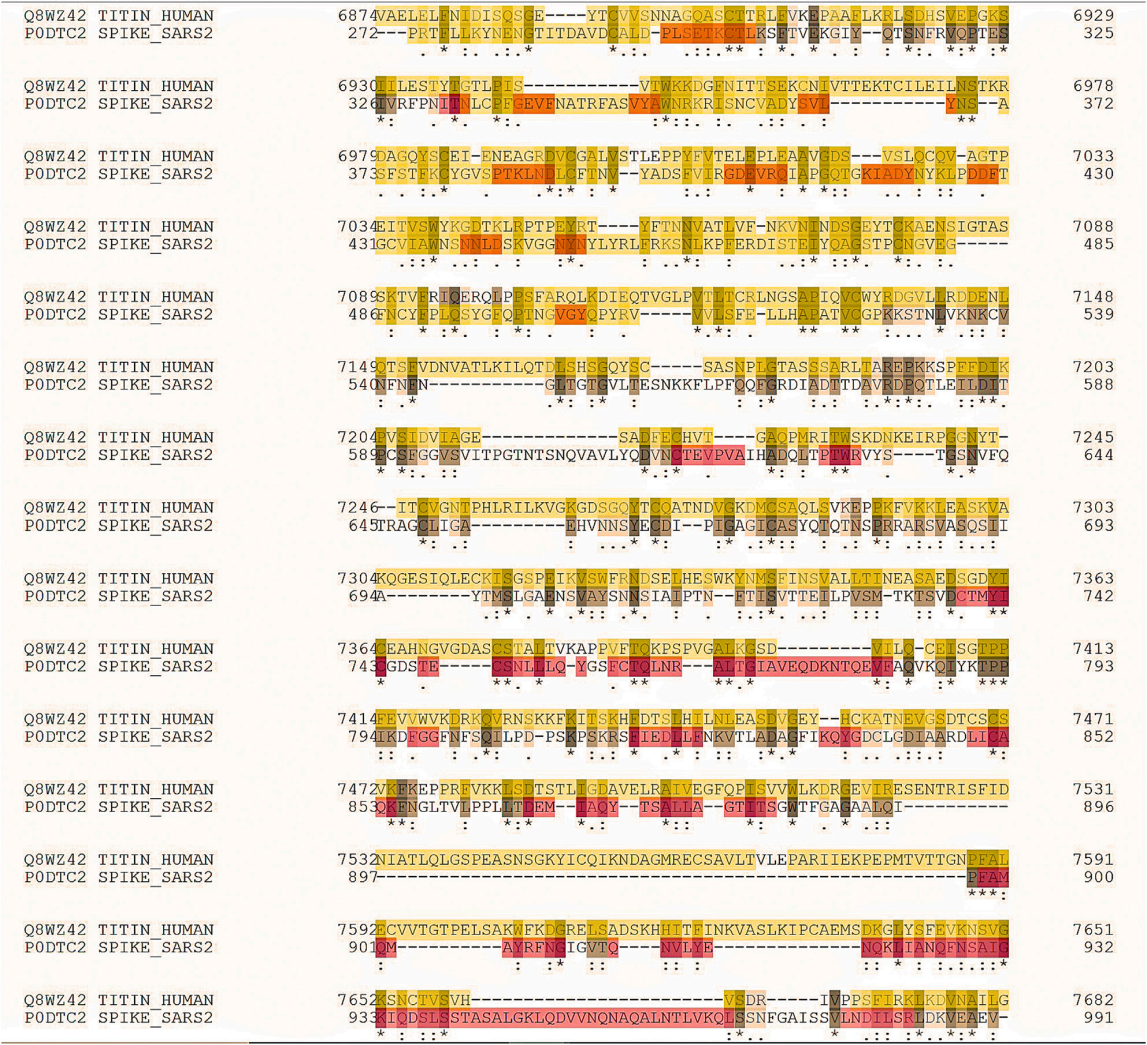
A20220109F248CABF64506F29A91F8037F07B67D1010CE6C (job identifier in UniProt) showing some of the 239 identical positions and some of the 428 similar positions. Proteomic analysis revealed a striking similarity between the SARS-CoV-2 spike glycoprotein (gp) and the cardiac human protein titin.

In June 2021, the rate of post-vaccine myocarditis was significantly higher than the expected rate. Rarely, the associated myocardial tissue changes have been satisfactorily evaluated with the degree of cardiac involvement seen in multisystemic inflammatory syndrome in children (MIS-C), which remains exceedingly rare. The 2018 revised Lake Louise cardiovascular magnetic resonance (CMR) criteria are difficult to set up worldwide. This is particularly true in low- and middle-income countries or high-income countries with limited resources. There is considerable speculation that myocarditis may be underreported in this age group. Titin is an essential human protein and the third most abundant striated-muscle protein. Two titin molecules span the sarcomere. They are anchored at the Z-line and M-line. Titin is necessary for the sarcomere assembly. It modulates the active contractile force and provides passive stiffness to cardiomyocytes (Chan et al., 2021). Doxorubicin (DXR), a cardiotoxic anticancer drug, enhances oxidative stress and stimulates cardiomyocytes’ matrix metalloproteinase-2 (MMP-2). Recently, we demonstrated that MMP-2 activation is an early event in DXR cardiotoxicity. It seems to contribute to myofilament lysis by proteolyzing the cardiac titin. Two orally available MMP inhibitors improved DXR cardiotoxicity. The intracellular and extracellular matrix remodeling attenuated, suggesting their use may be a potential prophylactic strategy to prevent heart injury during chemotherapy (Chan et al., 2021). It has been proved that anti-interleukin 6 (anti-IL-6) receptor antibodies enhance cardiac dysfunction and left ventricular remodeling in experimental myocarditis animal models using Coxsackievirus B3 (CVB3). Compared to controls, the infected mice demonstrated a decreased function of the left ventricle (systolic and diastolic), which was associated with increased inflammation, fibrosis, and impaired titin phosphorylation. The IL-6 receptor blockade constantly led to a shift of the immunologic response to a Th1 direction and significant viral load reduction. The IL-6 receptor blockade utilizes favorable cardiac effects. Antiviral actions and immunomodulatory activities mediate this effect. In numerous cases of acute myocarditis, titin and the cytoskeleton are dysregulated. The CMR-identified myocardial edema may be a substantial issue for children and young adults in the future due to a potentially dysregulated titin. Proteomic analysis revealed a striking similarity between the SARS-CoV-2 spike glycoprotein (gp) and the human cardiac protein titin. It has been calculated that 29 pentapeptides are shared between the two proteins (Kanduc, 2021). The peptide commonality does not reflect mere peptide sharing. Instead, it shows relevant immunological potential. Nearly all shared sequences are present in experimentally validated SARS-CoV-2 spike gp-derived epitopes, thus supporting the possibility that the virus can trigger anti-titin autoimmune disease cross-reactions (Figure 2). The peptide sharing between the two species may evoke immune responses that cross-react with human proteins, thus causing harmful autoimmunity (Sergi, 2022b).

Conversely, liver reactions seem much less frequent than myocarditis, pericarditis, or “cardiac discomfort.” The current downdegradation of SARS-CoV-2 to a mild symptomatic or minimal symptomatic or, even, asymptomatic stage has allowed to cool down the animosity and read the data more cautiously, specifically now that Pfizer Inc., one of the mRNA technology-based vaccine, is revisiting some protocols and the contract with European countries. According to Reuters and the Financial Times, Pfizer Inc. has recently agreed to extend its COVID-19 vaccine contract from 2023 to 2026 with the European Union. In addition, the company decided to decrease the number of doses needed to be supplied by 40%. However, the situation has been heated up because Pfizer Inc. would reduce up to 0.5 billion COVID-19 inoculation doses the EU has committed to acquire this year in return for a more substantial higher price.

Autoimmune reactions are due to an immunologic intolerance against self-antigens, which is combined with an insufficiency of homeostatic mechanisms preventing a promiscuous immunologic response to these antigens. The mechanisms behind this failure may rely on the molecular mimicry as hypothesized for titin and myocarditis/pericarditis, but involving other antigenic determinants, which are chief to the formation of autoantibodies. Other theories include the building of immune complexes affecting T-lymphocyte balance and cell bystander activation as a consequence of a boisterous innate immunologic response to some molecular adjuvants that were added to most vaccines. Guillain–Barre syndrome and multiple sclerosis, two neurologic syndromes, have also been reported with it and have also been reported with other vaccination programs. Moreover, COVID-19 vaccination programs have been associated in numerous thromboembolic events. These happenings have been categorized as vaccine-induced thrombocytopenia or better pro-thrombotic immune thrombocytopenia (VIPIT). It has been reported that vaccine-promoted antibodies determine platelet activation. Consequently, there is thrombosis and then thrombocytopenia analogous to heparin-induced thrombocytopenia or idiopathic thrombocytopenic purpura or VIPIT.

Moreover, anti-titin antibodies have been found in a subset of patients with *myasthenia gravis*, a neuromuscular junction disease that is mostly associated with autoimmune antibodies, such as anti-acetylcholine receptor antibody (Romi, 2011). The co-existence of *myasthenia gravis* and autoimmune hepatitis is rare (Han et al., 2000; Mao et al., 2011), and in a cohort of patients with *myasthenia gravis* anti-titin antibodies seem to be relevant to autoimmunity (Szczudlik et al., 2014) in consideration of these data and the number of patients who may not be symptomatic.

The mRNA-based vaccine utilizes engineered RNA to satisfactorily code for an antigenic protein and generates intrinsic immunogenicity. An activation of cytokines with the pro-inflammatory characteristic before mRNA translation through binding of nucleotides with pattern-recognition receptors and recognition by toll-like receptors, leading to immune activation, might play a critical role in the development of immune-mediated diseases in patients receiving this type of vaccine. AIH, emerging after the inoculation of COVID-19 vaccination, is probably still a very rare event, and very few cases have been reported with clinical, biochemical, and histologic supportive data. These patients have been summarized by Chow et al. (2022).

Chow et al. identified 32 patients having COVID-19 vaccine-induced AIH-like syndrome (Chow et al., 2022). The patients had an average age of 55.6 years with a standard deviation (SD) of 15.3 years. More than two-thirds of the patients (68.8%) were women. With regard to ethnicity, six cases were Caucasian and 26 with an unspecified race and/or ethnicity. In 16 patients, the Pfizer-BioNTech vaccine was used, while 13 patients received the Moderna vaccine, and ultimately, three patients received the Oxford–AstraZeneca vaccine. Nine patients had a history of autoimmune disease, and five patients suffered from both liver and autoimmune diseases in the past medical history.

Adenovirus is infrequently associated with liver disease. There may be a feeble immunologic status as a probable life condition in some patients with adenovirus infection, as established by McKillop et al., in a patient with atypical teratoid rhabdoid tumor (McKillop et al., 2015). However, if adenovirus is a hypothesis for AHUO or liver disease during this COVID-19 pandemic, it does not fully elucidate the severity of the medical phenotype. The described infection with subtype 41 of adenovirus has not been associated with such a clinical picture or an AIH or AIH-like disease to the best of our knowledge. The immunocompetency of an individual is useful in avoiding a severe disease when encountering an adenovirus. Infections with adenovirus are commonly self-limited events in a substantially immunocompetent subject. These infections remain challenging to explain AHUO, and a simultaneous or coincidental event, in our opinion, cannot be ruled out.

Two important considerations are that AIH has been reported with several COVID-19 vaccines, as seen in Chow et al., who identified 32 patients having COVID-19 vaccine-induced AIH-like syndrome. The Janssen and Sinovac/CoronaVac vaccines have also been approved in several countries other than Pfizer and AstraZeneca. It remains speculative if the use of two doses (AstraZeneca) *vs.* one dose (Janssen) or the use of inactive adenovirus (Sinovac/CoronaVac) could affect the risk of development of AIH as a side effect. Moreover, it remains to be discussed that autoimmune responses after immunization with COVID-19 vaccines (including AIH) may not be reported satisfactorily due to testing costs. On the other hand, it is possible that all the side effects of the COVID-19 vaccines, particularly mRNA vaccines, are “over-reported” or reported utterly when compared to other vaccines due to all media attention that happened when the vaccination process started. Individuals vaccinated with COVID-19 vaccines have been much more attentive to the appearance of side effects, especially due to the temporary suspension of the AstraZeneca vaccine in response to reports of rare blood clotting events that happened in some countries. We should also concede that there are no clear clinical or biochemical features apart from a chronological association to differentiate patients’ vaccine-related AIH from idiopathic AIH, but it seems more than coincidental. We hope to raise awareness of likely side effects, following COVID-19 vaccination, and an increased role of pharmacovigilance is more than legitimate in guiding treatment. Only large, multicenter, longitudinal studies enrolling patients from across the globe can truly validate the findings of AIH or AIH-like reports, and watchfulness in healthcare is critical. On the other hand, coincidental or not, the current mild course of COVID-19 should stop the continuous unnecessary COVID-19 vaccination and the potential triggering of both autoimmune and pro-clotting events (Chow et al., 2022; Efe et al., 2022; Izagirre et al., 2022; Trontzas et al., 2022; Zheng et al., 2022; Gips and Woreta, 2023; Hartl et al., 2023). There is substantial evidence suggesting that we do not know how rare myocarditis/pericarditis and AIH are after COVID-19 mRNA vaccination without a proper animal model, as indicated previously (Sergi and Chiu, 2021). The isolation of a *Drosophila* gene using the serum from a patient with autoimmune scleroderma recognized an epitope on chromosomes (condensed mitotic form) in both human cultured cells and early *Drosophila* embryos. It revealed that this gene encodes a *Drosophila* homolog of the vertebrate titin (D-Titin) (Machado et al., 1998). In consideration of these data, we postulated that autoimmune phenomena may not be exceedingly rare, following the administration of mRNA technology-based vaccines, and a balance between pros and cons in administrating boosters is critical.

## Conclusion

In our opinion, underreporting is probably one major issue in both industrialized countries and countries with low and medium income. Physicians and families should be aware of this risk, which should be considered in subjects presenting with even minimal chest pain or increase in serum levels of liver biochemical values and autoantibodies within a week or two after vaccination. It is currently important to consider the true cost–benefit relationship for this kind of inoculation program in both children and young adults. MIS-C is frightful, but it is an uncommon event of COVID-19. The statement *“primum non nocere*,*”* which is translated as “first, do no harm,” should persist well-regarded in medicine.

## Data Availability

The original contributions presented in the study are included in the article/Supplementary Material. Further inquiries can be directed to the corresponding author.
